# Effect of vaginal flora on clinical outcome of frozen embryo transfer

**DOI:** 10.3389/fcimb.2022.987292

**Published:** 2022-12-12

**Authors:** Li Ji, Chen Peng, Xueyun Bao

**Affiliations:** ^1^ Center for Reproductive Medicine, Affiliated Hospital of Nantong University, Nantong, Jiangsu, China; ^2^ Department of Clinical Medicine, Nantong University, Nantong, Jiangsu, China

**Keywords:** infertility, sterility, *in vitro* fertilization, frozen embryo transfer, clinical outcome, vaginal flora, pregnancy rate, lactobacillus

## Abstract

**Objective:**

Vaginal microbiota imbalance is a high risk factor for premature birth, and is closely related to female pelvic inflammation and sexually transmitted diseases. The effect of vaginal microbiota on the outcome of assisted reproductive technology is still unclear. In this study, the vaginal microbial composition and the pregnancy outcome of frozen embryo transfer (FET) was investigated.

**Methods:**

From October 2020 to December 2021, 275 FET cycles were selected from the center of reproductive medicine in Affiliated Hospital of Nantong University. Vaginal secretions were collected on the day of endometrium transformation, and smears were Gram stained. According to the Nugent score they were divided into three groups, including normal group, mild dysbiosis group and sever dysbiosis group. The clinical outcomes of each group were compared.

**Results:**

In 275 FET cycles, the embryo implantation rate, clinical pregnancy rate and ongoing pregnancy rate in the normal group (66.9%,84.3% and 83.1%) were significantly higher than those in the mild dysbiosis group (45.5%, 57.3% and 49.3%) and in sever dysbiosis group (29.6%, 34.2% and 27%). The difference was statistically significant (*P*<0.01). When compared the preclinical pregnancy loss rate and the miscarriage rate, the normal group (1.3% and 1.3%) was significantly lower than those in the mild dysbiosis group (20.4% and 14.0%) and the sever dysbiosis group (25.5% and 21.1%). The difference was statistically significant (*P*<0.01), but there was no significant difference between the mild dysbiosis group and sever dysbiosis group (*P*>0.05).

**Conclusion:**

Nugent score is directly related to the clinical outcome of FET. The Lactobacilli-dominant vaginal flora was a favorable factor for the good clinical outcome of FET, while asymptomatic bacterial vaginosis had a negative correlation with the outcome of FET.

## Introduction

Frozen embryo transfer (FET) plays an increasingly important role in assisted reproductive technology (ART) (Singh et al., 2020). Children born *via* FET accounts for about 60% of total children born through ART (National Perinatal Epidemiology & Statistics Unit, 2022). With the wide application of vitrification cryopreservation technology, the resuscitation rate, implantation rate and clinical pregnancy rate of frozen embryos have achieved satisfactory clinical outcomes (Loutradi et al., 2008). However, the live birth per FET was only 23.4% (National Perinatal Epidemiology & Statistics Unit, 2022). The reasons of the failure of embryo implantation are complicated. In addition to embryo quality and endometrial receptivity, the two main factors of the success of embryo implantation, there are still many problems to be explored (Franasiak et al., 2021). With the deepening of the research on human microbiota, the mysterious veil of the relationship between microorganism and disease has been gradually unveiled (Blum, 2017). In the field of reproduction, relationship between microbiota of reproductive tract and reproductive health has also attracted much attention (Anahtar et al., 2018).

Microbes live with us and make up about 1-3% of our body weight. Studies on reproductive tract microbes have found that the vaginal microbiota played a key role in maintaining the physiological balance of the vaginal environment and avoiding the invasion of conditioned pathogens (Lewis et al., 2017). *Lactobacillus* is dominant in the vaginal microbiota of healthy women, and most women have only one *Lactobacillus* genus, such as *L. Crispatus*, *L. Iners*, *L. Jensenii* and *L. Gasseri* (Xu et al., 2020). *Lactobacillus* not only inhibits the production of pathogenic bacteria, but also reduces vaginal pH by producing lactic acid and hydrogen peroxide (Ahrens et al., 2020). Vaginal acidic environment and hydrogen peroxide play an important role in resisting the invasion and reproduction of anaerobic bacteria and opportunistic pathogens (Gong et al., 2014). The disorder of vaginal fluid causes the imbalance of vaginal environment and lead to the excessive growth and reproduction of pathogenic microorganisms. Bacterial vaginosis (BV) is the most common vaginal disease in women of childbearing age (Abou Chacra et al., 2022). The vaginal microbiota of the patients changes from the dominant *Lactobacillus* to a highly complex multi-bacterial community, including the mixed infection of *Gardnerella* vaginalis and some anaerobic bacteria, producing a variety of biogenic amines and short-chain fatty acids (Ceccarani et al., 2019). Clinical manifestations of BV include vaginal discharge, itching, and burning. However, approximately 50% of BV patients are asymptomatic (Van Oostrum et al., 2013), and the effect of vaginal fluid in these patients on infertility treatment remains to be investigated.

The disorder of vaginal microecological environment is associated with pelvic inflammatory disease, endometritis, preterm birth, abortion, infertility and sexually transmitted infection (Juliana et al., 2020), but its relationship with the pregnancy outcome of *in vitro* fertilization - embryo transfer (IVF-ET) pregnancy outcome is still unclear. The purpose of this study is to assess the composition of the to assess the composition of the vaginal microorganisms in FET women by Nugent score, and to explore the influence of vaginal microbiota on clinical outcomes of FET.

## Methods

### Patients recruitment

A total of 229 patients (include 275 cycles) who received FET from October 2020 to December 2021 in Center for Assisted Reproduction of Affiliated Hospital of Nantong University were selected. All participants were of childbearing age (29 ± 3 years old and less than 40). The presence of genital tract pathogens in vaginal/endocervical swabs was evaluated one month before FET. None of the patients had signs or symptoms of genital infection, such as neisseria gonorrhoeae, chlamydia trachomatis, mycoplasma humanum, candida vulva or trichomonas infection. Vaginal suppositories, antibiotics, and vaginal irrigation were not used within two weeks before sampling. Endometrial thickness was ≥7mm before transplantation. Exclusion criteria included uterine malformation, intrauterine adhesions, endometrial polyps, hydrosalpinx, thyroid dysfunction and immune diseases such as antiphospholipid syndrome. Written informed consent was obtained from each patient. The hospital ethical committee approved the study.

### Sample collection

For each patient, vaginal secretions were retained at the upper and middle 1/3 of the vagina using sterile cotton swabs and evenly applied on the sterile slide on the day of endometrial transformation. The smears were heat fixed and Gram stained.

### Vaginal microbiota evaluation

**Figure 1:**
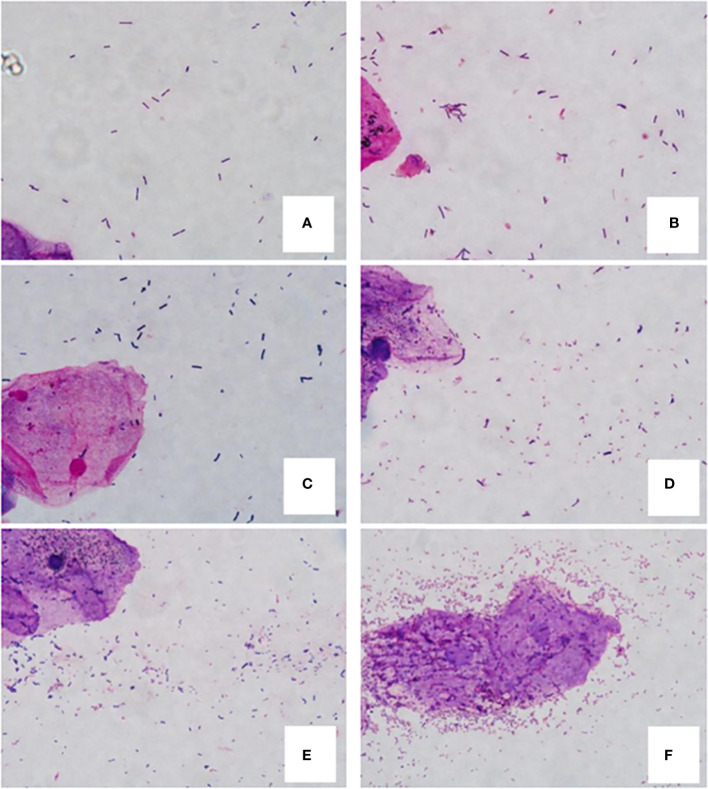
Gram-stained vaginal smears from women. **(A, B)** are normal flora samples. **(A)** 0 score (4+ *Lactobacillus* morphotypes, no small gram-negative or gram-variable rods), **(B)** 2 score (4+ *Lactobacillus* morphotypes, 2+ small gram-negative or gram-variable rods); **(C, D)** are mild dysbiosis vaginal flora samples. **(C)** 4 score (3+ *Lactobacillus* morphotypes, 3+ small gram-variable rods), **(D)** 6 score (2+ *Lactobacillus* morphotypes, 4+ small gram-negative and -variable rods); E and F are sever dysbiosis vaginal flora samples. **(E)** 7 score (3+ *Lactobacillus* morphotypes, 4+ gram-negative and -variable rods), **(F)** 10 score (no lactobacilli and 4+ gram-negative rods and curved rods, clue cell is in the center of field).

According to Nugent standard (Nugent et al., 1991), each Gram-stained smear was evaluated for the morphotypes under oil immersion (x1,000 magnification) by the same inspector. Each morphotype was quantitated from 0 to 4+ regarding the number of morphotypes in each oil immersion field (0, no morphotypes; 1+, less than 1 morphotype; 2+, 1 to 4 morphotypes; 3+, 5 to 30 morphotypes; 4+, 30 or more morphotypes) through double-blind method. Summing the weighted quantitation (0, 1 to 4+) of the various morphotypes, we get a score of 0 to 10 for each person. A score of 0 to 3 was considered normal, a score of 4 to 6 was considered mild dysbiosis, and a score of 7 or higher was considered sever dysbiosis (**Figure 1**).

### Endometrium preparation and FET

Endometrium was prepared through natural cycle or hormone replacement cycle. Transvaginal ultrasound and blood tests were performed. Natural FET cycles were used for women with regular menstrual cycles and began on day 10 or day 12 of menstruation. When the average diameter of the dominant follicles was greater than 17mm, the endometrial thickness was at least 7mm and the estradiol level was at least 150pg/mL, the trigger of ovulation was induced with 10000 IU of human chorionic gonadotropin ((hCG, Livzon Pharmaceutical Group Inc). Then dydrogesterone (Abbott Healthcare Products B.V.) tablets were administered orally (2 tablets, twice a day), and soft vaginal P capsules (Laboratoires Besins International) were administered (200mg, twice a day) as well. For hormone replacement cycle, it was recommended to take Estradiol Valerate (Bayer plc) orally, with the starting dose of 1mg, twice a day and, gradually increased to 4mg, twice a day from day 3 to day 14 of the cycle. When the endometrial lining of the patient was confirmed to be thicker than 8mm and the estradiol level was at least 150pg/mL, vaginal progesterone would be used as same as that in natural cycles. In case of inadequate endometrial thickness or insufficient estradiol level, vaginal estradiol or higher doses of oral estradiol was administered. Vitrified embryo freezing technology was used to freeze embryos. Embryos in the cleavage stage were thawed on day 3 after ovulation or endometrial transformation; while blastocysts were thawed on day 5. Transfers were performed under the guidance of abdominal ultrasound.

### Outcome assessment

Serum hCG was detected 10 days following embryo transfer. HCG higher than 5IU/L was considered positive. The primary outcome of the study was the clinical pregnancy rate per cycle. Secondary outcomes included the rates of implantation, preclinical pregnancy loss, miscarriage, and ongoing pregnancy. Clinical pregnancy was defined as the presence of an intrauterine gestational sac (with or without fetal heartbeat) by transvaginal ultrasound. The implantation rate was calculated by dividing the number of gestational sacs under ultrasound by the number of embryos transferred. Preclinical pregnancy loss was defined as a positive hCG that gradually dropped to <5 IU/L and without an intrauterine gestational sac. Ongoing pregnancy was defined as the presence of fetal heartbeat beyond 12 weeks of gestation. Miscarriage was defined as the loss of any form of pregnancy within the first 28 weeks of gestation.

### Statistical analysis

Statistical analysis was conducted with SPSS version 25. The descriptive statistics for the continuous (quantitative) variables are expressed as averages and standard deviations. One-way ANOVA was used for the comparison among multiple groups with normal distribution and equal variance. When the levene’s test showed that the variance was different, the Welch’s test was employed to compare the two variables for each group, Body Mass Index (BMI) and average number of embryos transferred. The Chi square (χ2) test or Fisher exact test was employed to assess the association of categorical variables, which were described as the frequency with rate, between the vaginal microbiota in vaginal swabs and the outcome of FET. *P*<0.05 was regarded as statistically significant.

## Results

A total of 275 cycles were included in the analysis. The average of patients’ ages was 30 years old, ranging from 20 to 39 years old. There were 190 cycles of primary infertility patients and 85 cycles of secondary infertility patients. There were 89 (32%) patients whose vaginal swabs microbiota scores were 0 to 3, 75 (27%) patients whose scores were 4 to 6, while 111 (40%) patients had vaginal microbiota sever dysbiosis (scores no less than 7). The demographic data and baseline characteristics in each group are shown in **Table 1**. No difference was found among normal, mild dysbiosis and sever dysbiosis groups in terms of age, duration of infertility, infertility type, cause of infertility, basic follicle-stimulating hormone (FSH) level, number of Antral Follicle Counting (AFC), endometrial thickness on the transfer day, embryo transfer stage and optimal embryo transfer rate (*P*>0.05). BMI and the average number of embryos transferred in each group were similarly adjusted by Welch’s test.

**Table 1:** Baseline characteristics of patients with different vaginal flora.

	Normal	Intermediate	Dysbiosis	P
Age(y)	30.1 ± 43.92	29.4 ± 3.45	29.7 ± 3.95	0.410
BMI(kg/m2)^a^	22.6 ± 3.49	23.5 ± 2.95	23.3 ± 3.72	0.174
Infertility duration(y)	4.37 ± 3.13	4.03 ± 2.81	4.05 ± 2.84	0.685
Infertility type (primary/secondary)	62/27	47/28	81/30	0.571
Infertility factor (n)				
Tubal factors	29	30	42	0.519
Ovulation obstacle	8	8	7	
Male factor	20	15	19	
Endometriosis	7	8	9	
Both factors	8	9	17	
Other	17	5	17	
rFSH(mIU/ml)	7.13 ± 2.46	7.08 ± 2.27	7.35 ± 2.26	0.676
AFC	26.7 ± 16.1	24.4 ± 15.8	23.9 ± 13.9	0.412
Endometrial thickness on transplantation day (mm)	9.76 ± 1.49	10.1 ± 1.61	10.0 ± 2.00	0.350
Average number of Embryos transferred a	1.60 ± 0.49	1.64 ± 0.48	1.52 ± 0.52	0.276
Phase of embryo transferred (cleavage embryo / blastocyst)	30/59	22/53	39/72	0.704
Excellent embryo rate of embryo transferred (%, n)	92.3(131/142)	87.0(107/123)	91.1(154/169)	0.318

^a^When the variance was different, the Welch’s test was employed to compare the two variables for each group. P < 0.05 was regarded as statistically significant.

Pregnancy outcomes grouped by vaginal microbiota are shown in **Table 2**. The significant differences of pregnancy outcomes among three groups were marked with an asterisk. We found significant differences of the rates of implantation (66.9% vs. 45.5% vs. 29.6%; *P*<0.001), clinical pregnancy (84.3% vs. 57.3% vs. 34.2%; *P*<0.001), ongoing pregnancy (83.1% vs. 49.3% vs. 27%; *P*<0.001), preclinical pregnancy loss (1.3% vs. 20.4% vs. 25.5%; *P*<0.001) and miscarriage (1.3% vs. 14% vs. 21.1%; *P*<0.01) among the three groups. Refer to implantation rate clinical pregnancy rate and ongoing pregnancy, the differences were significant between each two groups. For the rates of preclinical pregnancy loss and miscarriage, the differences between normal group and dysbiosis group are significant, while that between mild dysbiosis group and sever dysbiosis group are not.

**Table 2:** Clinical outcomes stratified by different vaginal microbiota patients.

	Normal	Intermediate	Dysbiosis	P	χ/ Fisher
Implantation rate % (n)	66.9 (95/142)^a^	45.5 (56/123^b^	29.6 (50/169)^c^	0.000^*^	43.26
Clinical pregnancy % (n)	84.3 (75)^d^	57.3 (43)^e^	34.2 (38)^f^	0.000^*^	50.39
Ongoing pregnancy % (n)	83.1 (74)^h^	49.3 (37)^i^	27.0 (30)^j^	0.000^*^	62.42
Pre-clinical pregnancy loss % (n)	1.3 (1/76)^l^	20.4 (11/54)^m^	25.5 (13/51)^m^	0.000^*^	17.76
Miscarriage % (n) R	1.3 (1/75)^n^	14.0 (6/43)^q^	21.1 (8/38)^q^	0.001^*^	12.57

* was regarded as statistically significant (P < 0.05). Abc, def, hij was regarded as statistically significant between every groups (P < 0.001), lm, nq was regarded as statistically significant between both groups (P < 0.01), mm, qq was regarded as there are not significance between both groups (P>0.05). R: Since more than 20% of the grids with theoretical number less than 5 are used, Fisher's exact test is adopted.

## Dissussion

The present study addresses, for the first time, the association between vaginal flora and clinical outcomes of FET. We found that the clinical pregnancy rate and ongoing pregnancy rate of the women dominated by *Lactobacillus* species after FET were significantly higher than those of women with a low percentage of *Lactobacillus*, and the early pregnancy loss occurred in dysbiosis group was significantly higher than that in *Lactobacillus*-dominated group. There have been multiple studies which indicated that vaginal microbiota was closely related to reproductive health (Smith and Ravel, 2017). Mycoplasma, Chlamydia trachomatis, candida vaginalis and Neisseria gonorrhoeae have been identified as risk factors for infertility (Tuddenham et al., 2022). Therefore, we routinely carried out the tests for these pathogens and treated all abnormal patients before *in vitro* fertilization (IVF) treatment to exclude vaginal infection factors leading to the reduction of pregnancy rate. Symptomatic BV patients are also treated for subsequent embryo transfer. However, insufficient emphasis has been placed on the impact of vaginal microbiota on IVF outcomes.

It was found that women who underwent IVF or IVF-ICSI treatment following fresh ET with a low percentage of *Lactobacillus* in their vaginal sample were less likely to have a successful embryo implantation using 16S rRNA gene sequencing (Koedooder et al., 2019). They found that an unfavorable microbiome profile showed a statistically significant change in the risk of failure to become pregnant (Koedooder et al., 2019). Although 16S rRNA can analyze dozens of vaginal bacteria, the specificity of qualitative detection was not high (Fichot and Norman, 2013). Moreover, high-throughput sequencing is time-consuming, technically challenging and costly (Fichot and Norman, 2013). There are also some other problems of high throughput sequencing, such as how to select a sequencing scheme and how to effectively analyze the massive data obtained by sequencing (Vos et al., 2012). Therefore, it is still not suitable for clinical application. Nugent score is the first standardized method based on the “morphological” classification and counting of Gram-stained vaginal smear, reflecting the composition and distribution of women’s vaginal microbiota to a certain extent (Nugent et al., 1991). Nugent score has many advantages, such as high sensitivity, strong specificity, easy to operate and inexpensive, so it is a reliable method and of great value for the evaluation of vaginal microbiota (Taylor-Robinson et al., 2003; Hogan et al., 2007). The vaginal bacterial communities of 40% (111/275) subjects showed persistently high Nugent scores, similar to that of BV subjects, although the subjects did not report any BV symptoms in this study. The possible reason for this phenomenon is that these women are in a state of subclinical vaginal dysbiosis, in which there is an overall decrease in *Lactobacillus* and an increase in non-*Lactobacillus* flora in vaginal secretions, but it has not yet reached the threshold for clinical symptoms.

The relationship between vaginal dysbiosis and pregnancy outcomes of ART has now begun to attract attention recently. The non-*Lactobacillus* bacterial species that have been found to be affected female reproductive health including *G. vaginalis species, Prevotella, Clostridia, phylum Fusobacteria, Class Fusobacteriia, order Fusobacteriales*, and *family Leptotrichiaceae* (Ruan et al., 2021). Vaginal dysbiosis is frequently associated with increased vaginal infections such as vulvovaginal candidiasis (VVC), cervicitis, gonorrhea and human immunodefciency virus (HIV) infection (Mitra et al., 2016; Van de Wijgert, 2017; Shannon et al., 2017; Eastment and McClelland, 2018). Previous studies have found association between vaginal dysbiosis and infertility using culture-dependent techniques (Fanchin et al., 1998; Selman et al., 2007). Pregnant women with vaginal dysbiosis are prone to occur poor outcomes, such as increased rates of early pregnancy loss (Eckert et al., 2003), increased tubal pregnancy (Ruan et al., 2021) and decreased pregnancy (Hyman et al., 2012). Hyman et al. (2012) found that *Lactobacillus*-dominant vaginal communities on the day of transplantation was an important factor for the success of IVF-ET, by examining the vaginal vault secretion of 30 IVF-ET patients with asymptomatic genital tract infection, and analyzing the influence of different hormone levels in IVF cycles on the composition of vaginal flora. Fu M et al. (Fu et al., 2020) analyzed the vaginal microbes of women with recurrent implantation failure (RIF) and found that the diversity increased, the abundance of anaerobic bacteria increased, and the abundance of *Lactobacillus* decreased. In this study, we found that the relationship between the composition of vaginal flora and the clinical outcome of FET cycle was similar to that of the fresh embryo transfer cycle in the above study. The clinical pregnancy rate and ongoing pregnancy rate decreased with the increase of Nugent score, while preclinical pregnancy loss and miscarriage were positively correlated with Nugent score. The results showed that *Lactobacillus* was not only beneficial to the clinical outcome of fresh embryo transplantation, but also beneficial to frozen embryo transplantation. The increase of vaginal microbial diversity not only reduced the clinical pregnancy rate of FET, but also was closely related to preclinical pregnancy loss and miscarriage.

In conclusion, we found a high correlation between Nugent score and pregnancy outcome of frozen embryo transplantation by taking vaginal microbiota as an independent factor in this study after excluding the influences of embryo, endometrium, pelvic environment, endocrine and other factors to minimize the interference. After decades of development, the pregnancy rate of assisted reproductive technology has been significantly improved. But the causes of embryo implantation failure are still unclear, and the mechanism of vaginal microbiota on embryo implantation or miscarriage needs further study. It is hoped that the management of reproductive tract infection in infertile couples can improve the clinical pregnancy rate, save the treatment cost and shorten the treatment time.

## Data availability statement

The original contributions presented in the study are included in the article/supplementary material. Further inquiries can be directed to the corresponding author.

## Ethics statement

The studies involving human participants were reviewed and approved by Ethics Committee of Affiliated Hospital of Nantong University. The patients/participants provided their written informed consent to participate in this study.

## Author contributions

CP: Substantial contributions to the conception or design of the work;provide approval for publication of the content;agree to be accountable for all aspects of the work in ensuring that questions related to the accuracy or integrity of any part of the work are appropriately investigated and resolved. LJ: Drafting the work or revising it critically for important intellectual content; XYB: the acquisition, analysis or interpretation of data for the work. All authors contributed to the article and approved the submitted version.

## References

[B1] Abou Chacra L. Fenollar F. Diop K. (2022). Bacterial vaginosis: What do we currently know? Front. Cell Infect. Microbiol. 11. doi: 10.3389/fcimb.2021.672429 PMC8805710 35118003

[B2] Ahrens P. Andersen L. O. Lilje B. Johannesen TB. Dahl EG. Baig S.. (2020). Changes in the vaginal microbiota following antibiotic treatment for mycoplasma genitalium, chlamydia trachomatis and bacterial vaginosis. PloS One 15 (7), e0236036. doi: 10.1371/journal.pone.0236036 32722712 PMC7386580

[B3] Anahtar M. N. Gootenberg D. B. Mitchell C. M. Kwon DS. (2018). Cervicovaginal microbiota and reproductive health: The virtue of simplicity. Cell Host Microbe 23 (2), 159–168. doi: 10.1016/j.chom.2018.01.013 29447695

[B4] Blum H. E. (2017). The human microbiome. Adv. Med. Sci. 62 (2), 414–420. doi: 10.1016/j.advms.2017.04.005 28711782

[B5] Ceccarani C. Foschi C. Parolin C. D'Antuono A. Gaspari V. Consolandi C.. (2019). Diversity of vaginal microbiota and metabolome during genital infections. Sci. Rep. 9 (1), 14095. doi: 10.1038/s41598-019-50410-x 31575935 PMC6773718

[B6] Eastment M. C. McClelland R. S. (2018). Vaginal microbiota and susceptibility to HIV. AIDS 32 (6), 687–698. doi: 10.1097/QAD.0000000000001768 29424773 PMC5957511

[B7] Eckert L. O. Moore D. E. Patton D. L. Agnew K. J. Eschenbach D. A. (2003). Relationship of vaginal bacteria and inflammation with conception and early pregnancy loss following in-vitro fertilization. Infect. Dis. Obstet Gynecol. 11 (1), 11–17. doi: 10.1155/S1064744903000024 12839628 PMC1852261

[B8] Fanchin R. Harmas A. Benaoudia F. Lundkvist U. Olivennes F. Frydman R. (1998). Microbial flora of the cervix assessed at the time of embryo transfer adversely affects *in vitro* fertilization outcome. Fertil Steril. 70 (5), 866–870. doi: 10.1016/s0015-0282(98)00277-5 9806568

[B9] Fichot E. B. Norman R. S. (2013). Microbial phylogenetic profiling with the pacific biosciences sequencing platform. Microbiome 1 (1), 10. doi: 10.1186/2049-2618-1-10 24450498 PMC3971627

[B10] Franasiak J. M. Alecsandru D. Forman E. J. Gemmell L. C. Goldberg J. M. Llarena N.. (2021). A review of the pathophysiology of recurrent implantation failure. Fertil Steril. 116 (6), 1436–1448. doi: 10.1016/j.fertnstert.2021.09.014 34674825

[B11] Fu M. Zhang X. Liang Y. Lin S. Qian W. Fan S. (2020). Alterations in vaginal microbiota and associated metabolome in women with recurrent implantation failure. mBio 11 (3), e03242–e03219. doi: 10.1128/mBio.03242-19 32487762 PMC7267891

[B12] Gong Z. Luna Y. Yu P. Fan H. (2014). Lactobacilli inactivate chlamydia trachomatis through lactic acid but not H2O2. PloS One 9 (9), e107758. doi: 10.1371/journal.pone.0107758 25215504 PMC4162611

[B13] Hogan V. K. Culhane J. F. Hitti J. Rauh V. A. McCollum K. F. Agnew K. J. (2007). Relative performance of three methods for diagnosing bacterial vaginosis during pregnancy. Matern Child Health J. 11 (6), 532–539. doi: 10.1007/s10995-007-0205-4 17874288

[B14] Hyman R. W. Herndon C. N. Jiang H. Palm C. Fukushima M. Bernstein D.. (2012). The dynamics of the vaginal microbiome during infertility therapy with *in vitro* fertilization-embryo transfer. J. Assist. Reprod. Genet. 29 (2), 105–115. doi: 10.1007/s10815-011-9694-6 22222853 PMC3270134

[B15] Juliana N. C. A. Suiters M. J. M. Al-Nasiry S. Morré S. A. Peters R. P. H. Ambrosino E. (2020). The association between vaginal microbiota dysbiosis, bacterial vaginosis, and aerobic vaginitis, and adverse pregnancy outcomes of women living in Sub-Saharan Africa: A systematic review. Front. Public Health 8. doi: 10.3389/fpubh.2020.567885 PMC7758254 33363078

[B16] Koedooder R. Singer M. Schoenmakers S. Savelkoul P. H. M. Morré S. A. de Jonge J. D.. (2019). The vaginal microbiome as a predictor for outcome of *in vitro* fertilization with or without intracytoplasmic sperm injection: a prospective study. Hum. Reprod. 34 (6), 1042–1054. doi: 10.1093/humrep/dez065 31119299

[B17] Lewis F. M. T. Bernstein K. T. Aral S. O. (2017). Vaginal microbiome and its relationship to behavior, sexual health, and sexually transmitted diseases. Obstet Gynecol. 129 (4), 643–654. doi: 10.1097/AOG.0000000000001932 28277350 PMC6743080

[B18] Loutradi K. E. Kolibianakis E. M. Venetis C. A. Papanikolaou EG. Pados G. Bontis I.. (2008). Cryopreservation of human embryos by vitrification or slow freezing: a systematic review and meta-analysis. Fertil Steril. 90 (1), 186–193. doi: 10.1016/j.fertnstert.2007.06.010 17980870

[B19] Mitra A. MacIntyre D. A. Marchesi J. R. Lee YS. Bennett P. R. Kyrgiou M. (2016). The vaginal microbiota, human papillomavirus infection and cervical intraepithelial neoplasia: what do we know and where are we going next? Microbiome 4 (1), 58. doi: 10.1186/s40168-016-0203-0 27802830 PMC5088670

[B20] National Perinatal Epidemiology & Statistics Unit (2022). Australia And new Zealand assisted reproduction database (Sydney: University of New South Wales).

[B21] Nugent R. P. Krohn M. A. Hillier S. L. (1991). Reliability of diagnosing bacterial vaginosis is improved by a standardized method of gram stain interpretation. J. Clin. Microbiol. 29 (2), 297–301. doi: 10.1128/jcm.29.2.297-301.1991 1706728 PMC269757

[B22] Ruan X. F. Zhang Y. X. Chen S. Liu X. R. Zhu F. F. Huang YX.. (2021). Non-Lactobacillus-Dominated vaginal microbiota is associated with a tubal pregnancy in symptomatic Chinese women in the early stage of pregnancy: A nested case-control study. Front. Cell Infect. Microbiol. 11. doi: 10.3389/fcimb.2021.659505 PMC8294389 34307190

[B23] Selman H. Mariani M. Barnocchi N. Mencacci A. Bistoni F. Arena S.. (2007). Examination of bacterial contamination at the time of embryo transfer, and its impact on the IVF/pregnancy outcome. J. Assist. Reprod. Genet. 24 (9), 395–399. doi: 10.1007/s10815-007-9146-5 17636439 PMC3454954

[B24] Shannon B. Gajer P. Yi T. J. Ma B. Humphrys M. S. Thomas-Pavanel J.. (2017). Distinct effects of the cervicovaginal microbiota and herpes simplex type 2 infection on female genital tract immunology. J. Infect. Dis. 215 (9), 1366–1375. doi: 10.1093/infdis/jix088 28201724 PMC5451606

[B25] Singh B. Reschke L. Segars J. Baker V. L. (2020). Frozen-thawed embryo transfer: the potential importance of the corpus luteum in preventing obstetrical complications. Fertil Steril. 113 (2), 252–257. doi: 10.1016/j.fertnstert.2019.12.007 32106972 PMC7380557

[B26] Smith S. B. Ravel J. (2017). The vaginal microbiota, host defence and reproductive physiology. J. Physiol. 595 (2), 451–463. doi: 10.1113/JP271694 27373840 PMC5233653

[B27] Taylor-Robinson D. Morgan D. J. Sheehan M. Rosenstein I. J. Lamont R. F. (2003). Relation between gram-stain and clinical criteria for diagnosing bacterial vaginosis with special reference to gram grade II evaluation. Int. J. STD AIDS. 14 (1), 6–10. doi: 10.1258/095646203321043183 12590785

[B28] Tuddenham S. Hamill M. M. Ghanem K. G. (2022). Diagnosis and treatment of sexually transmitted infections: A review. JAMA 327 (2), 161–172. doi: 10.1001/jama.2021.23487 35015033

[B29] Van de Wijgert J. H. H. M. (2017). The vaginal microbiome and sexually transmitted infections are interlinked: Consequences for treatment and prevention. PloS Med. 14 (12), e1002478. doi: 10.1371/journal.pmed.1002478 29281632 PMC5744905

[B30] Van Oostrum N. De Sutter P. Meys J. Verstraelen H. (2013). Risks associated with bacterial vaginosis in infertility patients: a systematic review and meta-analysis. Hum. Reprod. 28 (7), 1809–1815. doi: 10.1093/humrep/det096 23543384

[B31] Vos M. Quince C. Pijl A. S. de Hollander M. Kowalchuk G. A. (2012). A comparison of rpoB and 16S rRNA as markers in pyrosequencing studies of bacterial diversity. PloS One 7 (2), e30600. doi: 10.1371/journal.pone.0030600 22355318 PMC3280256

[B32] Xu J. Bian G. Zheng M. Lu G. Chan W-Y. Li W.. (2020). Fertility factors affect the vaginal microbiome in women of reproductive age. Am. J. Reprod. Immunol. 83 (4), e13220. doi: 10.1111/aji.13220 31925865 PMC7078941

